# Recent evolution in use and effectiveness in mainland China of thoracic endovascular aortic repair of type B aortic dissection

**DOI:** 10.1038/s41598-017-17431-w

**Published:** 2017-12-11

**Authors:** Jiang Xiong, Chen Chen, Zhongyin Wu, Duanduan Chen, Wei Guo

**Affiliations:** 1 Department of Vascular and Endovascular Surgery, Chinese PLA General Hospital, Beijing, P.R. China; 2 Department of Health Policy and Management, Jiann-Ping Hsu College of Public Health, Georgia Southern University, Statesboro, GA USA; 3 Department of Vascular Surgery, Affiliated Hospital of Chengde Medical College, Chengde, Hebei P.R. China; 4 Department of Biomedical Engineering, School of Life Science, Beijing Institute of Technology, Beijing, P.R. China

## Abstract

A meta-analysis was performed on 175 studies selected among those published in mainland China between 2008 and 2015 on thoracic endovascular aortic repair (TEVAR) for type B aortic dissection (AD). Most TEVAR were performed in Shanghai, Beijing, Hubei and Guangdong in patients with mean age of 53.9 years, and acute (70%) or chronic (30%) type B AD. Procedural success rate was 99.1 ± 0.8%. Major complication rate was 1.7 ± 2.3%, with paraplegia in 0.4 ± 0.0%. Overall in-hospital mortality rate was 1.6 ± 0.9% with AD rupture in 30% (about 40% during first postoperative day); follow-up mortality rate was 2.3 ± 1.1%, with AD rupture in 39.2% (50% within first year). Compared with 2001–2007 data from China, there appeared to be improvement in rates of major complications, paraplegia and in-hospital mortality. Compared with 1999–2004 Western data, rates of procedural success, stroke, and paraplegia appeared similar, while those for major complications, in-hospital mortality, retrograde type A dissection and follow-up mortality appeared lower. Compared with more recent Western data (2006–2013) on acute complicated type B AD, stroke, paraplegia, in-hospital mortality and follow-up mortality appeared lower. Therefore, in mainland China, safety for TEVAR of type B AD appeared better between 2008 and 2015 than in previous periods in China or Western countries.

## Introduction

Thoracic endovascular aortic repair (TEVAR), which was introduced in 1998 as a less invasive treatment option for type B aortic dissection (AD), is now widely used in China^1^. A meta-analysis by our group in 2009 on studies published from 2001 to 2007 including over 1300 patients documented that effectiveness of TEVAR for type B AD in mainland China compared favorably with that in Western countries (1999–2004)^2^. To gain insight into temporal evolution of clinical outcomes, baseline characteristics and use of TEVAR among type B AD patients in mainland China, the present meta-analysis summarized multicenter data published in 2008–2015 and compared them to previous periods in China and Western countries.

## Materials and Methods

### Data sources and study selection

“Aorta,” “dissection” and “stent” were used as key words for a comprehensive search of both English- and Chinese-language literature and postgraduate theses in PubMed, Embase, CNKI (Chinese National Knowledge Infrastructure) and CQVIP databases. All Chinese studies on TEVAR published between January 2008 and October 2015 were identified for further analysis. Inclusion criteria were: (1) articles including patients with type B AD undergoing retrograde endovascular stent-graft placement into the descending thoracic aorta; and (2) a series of at least 5 patients with type B AD who underwent TEVAR. Exclusion criteria were: (1) reports on antegrade, surgical (“open”) stent-graft placement through the aortic arch; (2) case reports; (3) reports on traumatic type B AD, iatrogenic injury, penetrating aortic ulcer or intramural hematoma; (4) reports on connective tissue disease (e.g., Marfan syndrome or Ehlers-Danlos syndrome); and (5) reports on other thoracic aortic pathologies (e.g., aneurysms).

### Data extraction and statistical analysis

Each article was analyzed using a standardized protocol with 35 predefined variables including clinical characteristics, procedural data, and in-hospital and long-term outcomes (Table S1). The statistical outcomes and figures were generated using STATA 12 software (StataCorp, College Station, Texas, USA) and Prism 6.0 software (GraphPad Software, Inc, CA, USA). Unspecified information was classified as unavailable and was not used for meta-analysis.

Rates of events were calculated as number of events divided by number of treated patients with available data. A weighted average estimate was calculated by combining individual study results. Weight assigned to each individual study was proportional to its sample size.

Results are presented as proportion, median with range or mean with standard deviation, as appropriate. The distribution of cases by geographic area (Chinese province/autonomous regions/municipalities) and medical specialty performing TEVAR (cardiology; vascular surgery; cardiothoracic surgery; interventional radiology; and general surgery) was described, analyzed and compared with the results from the aforementioned meta-analysis on studies published between January 2001 to December 2007 (Chinese data 2001–2007)^2^, as well as from the representative meta-analysis by Eggebrecht *et al*. of European and North American studies published between January 1999 and May 2004^3^. Western, and 2001–2007 and 2008–2015 Chinese data were compared using 2-sided Student *t* test. Kaplan–Meier survival curves were used to estimate in-hospital/30-day and long-term death. Rates of in-hospital/30-day procedure-related death and long-term death of patients who underwent TEVAR were compared with that of non–procedure related death by using the log-rank test. A *p* < 0.05 was considered statistically significant. Causes of in-hospital/30-day and long-term mortality were described.

### Data availability

All relevant data are within the paper.

## Definitions

Type B AD was classified based on the Stanford classification^4^. Dissection was considered as an acute event if occurring within the first 14 days after symptom onset, and chronic if beyond 14 days. Complications were classified as either major when life-threatening or prompting major therapeutic interventions (e.g., access complications requiring surgical revision), or minor when not requiring further treatment (e.g., transient renal failure not requiring dialysis). Stent-graft implantation was based on indications described by Nienaber and colleagues^5^, although some, such as distance between landing zone not <1.5 cm, maximal aortic diameter >5.5 cm, and no bilateral iliac tortuosity and stenosis, were not followed because of more positive attitude about TEVAR^6,7^, accumulating experience and device improvement^8,9^. For left subclavian artery (LSA) revascularization, use of surgical debranch or chimney technique was at operator’s discretion before intentional LSA coverage. Technical details of TEVAR were as described by Nienaber and colleagues^5^. Procedural success was defined as the technically successful deployment of the stent-graft at the intended target location. Any death that occurred suddenly or could not be related to other causes was defined as aortic rupture related death. Reintervention was defined as the need for any surgical conversion or additional endovascular stent-graft procedures. Procedure-related deaths refer to those related to endovascular stent placement (e.g., retrograde dissection, organ or peripheral artery malperfusion, or dissection rupture). Non–procedure-related deaths refer to those (e.g., myocardial infarction, cancer or multiple organ failure) unrelated to endovascular stent-graft placement.

## Results

### Study Selection

**Figure 1:**
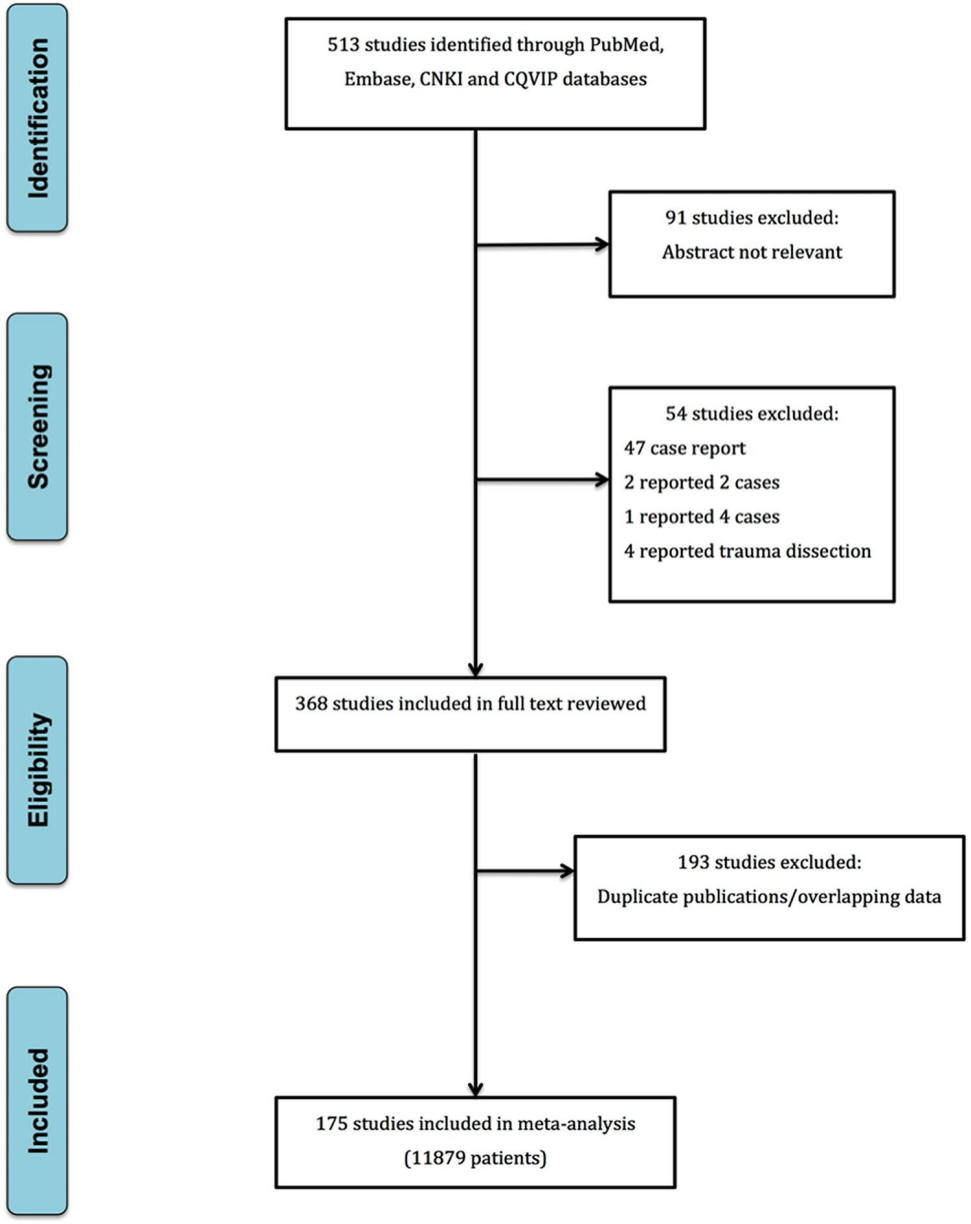
Flowchart of article inclusion and exclusion.

After excluding 91 studies with non-relevant abstract and 54 studies with <5 cases reported or traumatic dissection reports, 368 studies were selected for further full text review. Among the latter, 193 were excluded because of duplicate publications or overlapping data, and 175 studies were eventually selected for data extraction and analysis (supplemental references). A total of 11879 patients who underwent TEVAR were included in this meta-analysis (Fig. 1).

### Study and Patient Characteristics

An overview of study and patient characteristics is presented in Table 1. A total of 173 hospitals and 176 departments were included in the meta-analysis. Among patients with pertinent information: average age (among 8,580 patients) was 53.9 years; 70.6% (among 7,677 patients) were male; 70.2% (among 4,890 patients) had acute dissection; 83.5% (among 6,693 patients) had hypertension; and 5.4% (among 3,402 patients) had diabetes.

**Table 1:** Overview of study and patient characteristics.

	Data available (n)	No. of events or cases
Total No. of studies included	175	
Total No. of hospitals included	173	
Total No. of departments included	176	
No. of patients with type B AD	11879	
Average No. of patients with type B AD per study	67*	36^#^ (5–674)
Patient age (y)	8580	53.9*
Male sex	7677	6184 (80.6 ± 0.5%)
Acute dissection	4890	3432 (70.2 ± 4.4%)
Hypertension	6693	5591 (83.5 ± 0.6%)
Diabetes	3402	185 (5.4 ± 0.0%)

*Average, ^#^Median, AD: aortic dissection.

**Figure 2:**
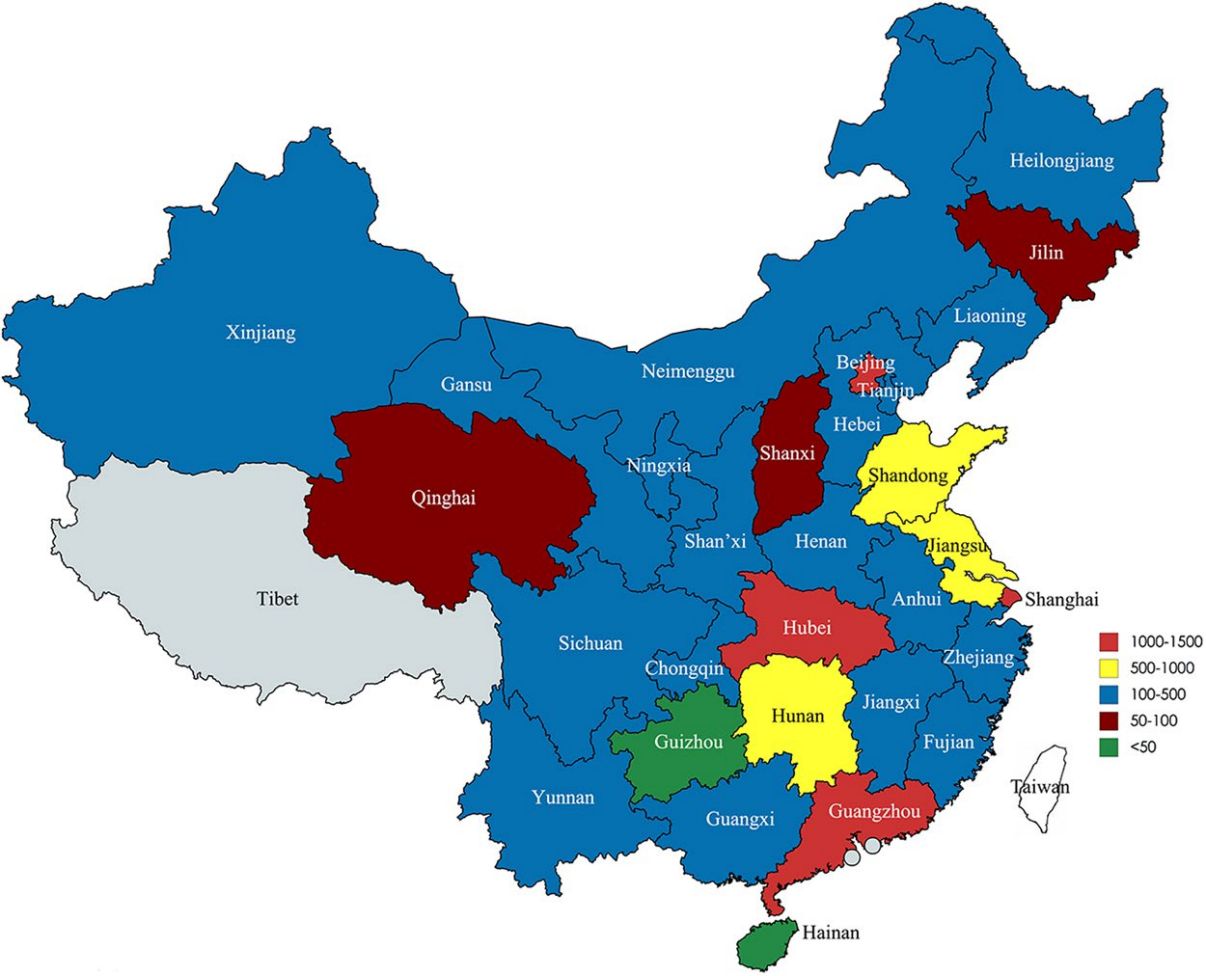
Geographical distribution of TEVAR cases in 30 administrative regions of mainland China (Jan. 2008–Oct. 2015). Number of cases was categorized into 5 levels (green: <50, brown: 50–<100, blue: 100–<500, yellow: 500–<1000, red: 1000–1500). This map is created using the “mapchart” online figure generator (https://mapchart.net/).

Numbers of included studies, patients, hospitals and departments by geographic area are shown in Table 2. Two municipalities and 4 provinces (Beijing, Shanghai, Zhejiang, Yunnan, Liaoning and Hunan) had over 100 patients per department. Among departments with published cases, the department of vascular surgery from Zhongshan Hospital in Shanghai had the largest number (443) of type B AD cases. Figure 2A shows TEVAR case prevalence by geographic region. Number of cases was categorized into 5 levels (green: <50, brown: 50- <100, blue: 100- <500, yellow: 500- <1000, and red: 1000–1500). Except for Tibet, all 22 provinces, 4 autonomous regions and 4 municipalities reported on TEVAR for type B AD. Two municipalities and 2 provinces (Shanghai, Beijing, Hubei and Guangdong) had 1000–1500 cases; three provinces had 500- <1000 cases; eleven provinces, 4 autonomous regions and 2 municipalities had 100–<500 cases; 2 provinces had 50– < 100 cases; and 2 provinces had <50 cases. Figure S1 presents the geographic distribution of cases in the 2001–2007 Chinese data meta-analysis: only 10 provinces, 4 autonomous regions and 2 municipalities reported TEVAR among type B AD patients, and the reported number of cases was only about one-tenth of that between 2008–2015; 2 municipalities and 1 province had 100–<500 cases; 2 provinces and 1 autonomous region had 50–<100 cases; 7 provinces and 3 autonomous regions had <50 cases; and the remaining 12 provinces, 1 autonomous region and 2 municipalities had no studies reporting TEVAR. The proportion of cases treated by each specialist type was calculated (Fig. S2A) and compared with 2001–2007 Chinese data (Fig. S2B). Vascular surgery had the highest (~40%) proportion of cases. When compared with 2001–2007 Chinese data, the proportion of cases in cardiology and cardiothoracic surgery both increased by over 5%, while that in interventional radiology decreased by 13%. Cases treated by general surgery were not reported in 2001–2007, and only accounted for a very small proportion (0.74%) in 2008–2015.

**Table 2:** Number of patients, studies, hospitals and departments by geographic area (Chinese mainland provinces/autonomous regions/municipalities).

Provinces, autonomous regions and municipalities	Patients (n)	Studies (n)	Hospitals (n)	Departments (n)	Patients per Department (n)
Xinjiang	352	5	5	5	70
Beijing	1281	9	8	8	160
Guangxi	225	7	7	7	32
Anhui	224	7	5	6	37
Jiangsu	757	15	15	15	50
Shan’xi	410	4	5	5	82
Qinghai	57	2	2	2	29
Sichuan	245	5	5	5	49
Hebei	215	5	5	5	43
Neimengu	110	5	4	4	28
Jiangxi	326	6	5	6	54
Chongqin	161	3	3	3	54
Shandong	668	13	13	13	51
Zhejiang	402	3	3	3	134
Fujian	186	4	4	4	47
Heilongjiang	145	4	4	4	36
Gansu	157	5	4	4	39
Tianjin	184	3	3	3	61
Henan	465	10	9	10	47
Guangdong	1051	16	20	20	53
Yunnan	453	4	4	4	113
Ningxia	118	2	2	2	59
Liaoning	411	4	4	4	103
Hunan	633	4	4	4	158
Hubei	1066	13	13	13	82
Guizhou	6	1	1	1	6
Shanghai	1428	9	9	9	159
Jilin	72	4	4	4	18
Shanxi	56	2	2	2	28
Hainan	15	1	1	1	15
Total	11879	175	173	176	67

### Procedural Data and In-Hospital Course

**Figure 3:**
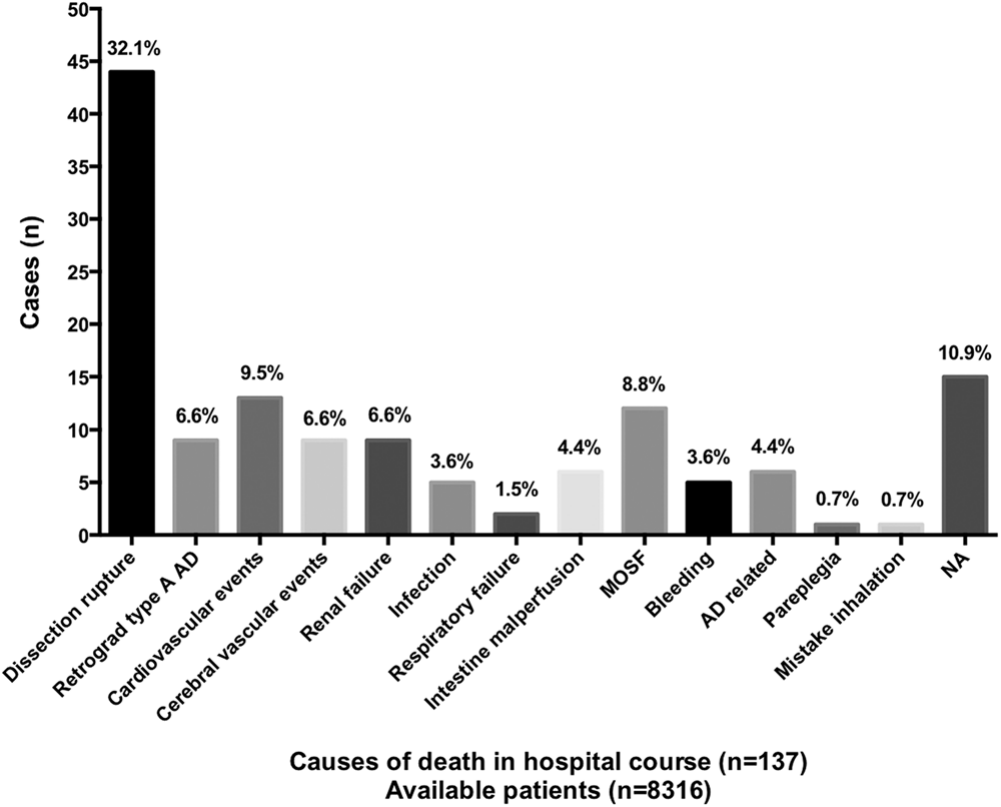
Types and proportions of in-hospital death causes. AD: aortic dissection, MOSF: multiple organ system failure, NA: not applicable.

**Figure 4:**
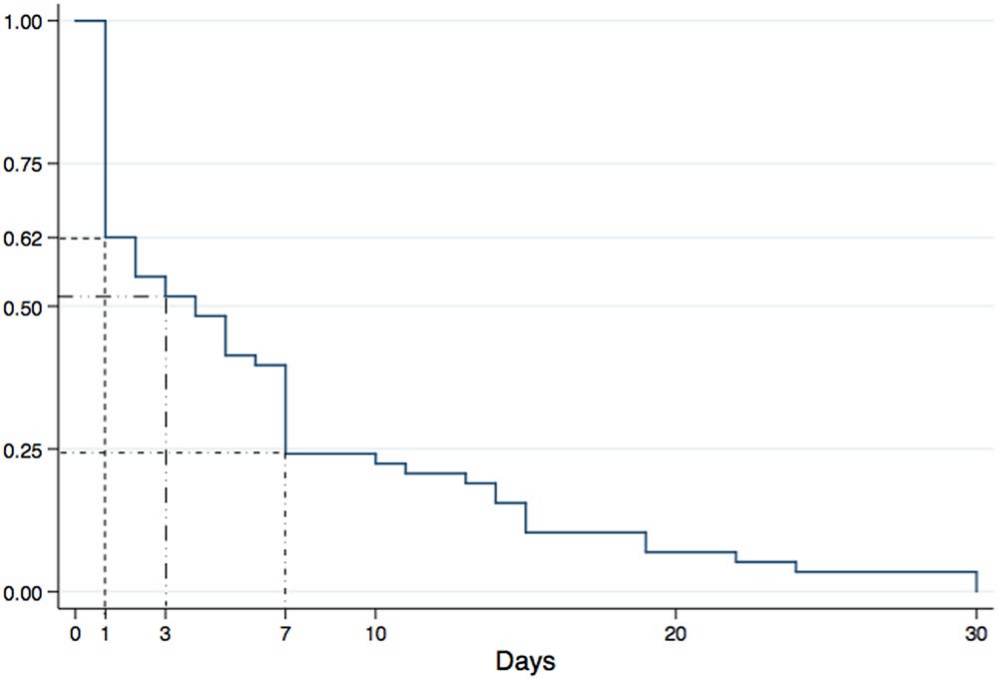
Kaplan-Meier survival curve for in-hospital death. First day deaths accounted for about 40% (dot line), death within the first 3 postoperative days accounted for about 50% (long dash-dot-dot line), and deaths within the first postoperative week accounted for about 75% (short dash-dot line) of in-hospital deaths.

Table 3 shows procedural data and in-hospital course. The majority of patients (71.8% among 5314) received general anesthesia. TEVAR was successful in 99.1% of 8885 patients. Regarding adjunctive techniques in TEVAR, covered left subclavian artery was used in 9.5%; debranch technique in 2.8% (among 8885 patients); and chimney technique in 6.1% among 783 patients. In-hospital major complications were reported in 1.7% of 6956 cases. The most critical in-hospital complications were related to retrograde dissection extension into the ascending aorta (0.4% of 6532 cases) and stent-graft related distal AD (0.1% of 6532 cases). In terms of neurologic complications, in-hospital paraplegia occurred in 0.4% of 1106 cases, and stroke occurred in 1.1% of 3695. Total in-hospital surgical conversion rate was 0.3% of 8885 cases. In-hospital adjunctive endovascular procedure was required in 0.3% of 8885 cases. One hundred and thirty-seven of 8316 cases died during the in-hospital period, yielding an overall in-hospital mortality rate of 1.6%. Type and proportion of in-hospital death causes are summarized in Fig. 3. Dissection rupture was the most common cause of death (32.1%). Among the 22 dissection rupture patients with known time of death, 36.4% died during the first postoperative day and 95.5% within postoperative week one. Figure 4 shows the Kaplan-Meier curve for in-hospital death among patients with available data. Forty percent of deaths occurred during the first postoperative day, 50% within the first 3 postoperative days, and ~75% within the first postoperative week. Figure S3 depicts rates of procedure- and non–procedure-related deaths during hospitalization, which were statistically similar (0.8% vs. 0.6%, log-rank p = 0.765).

**Table 3:** Procedural data and in-hospital course.

	Data available (n)	No. of events [n(% ± SE)]
Anesthesia	5314	
General anesthesia		3816 (71.8 ± 1.0%)
Local anesthesia		1283 (24.1 ± 2.9%)
Lumber anesthesia		22 (0.4 ± 0.0%)
Epidural anesthesia		174 (3.3 ± 0.0%)
Procedure success	8885	8809 (99.1 ± 0.8%)
Covered left subclavian artery	8885	843 (9.5 ± 1.9%)
Debranch	8885	248 (2.8 ± 0.9%)
Chimney	783	48 (6.1 ± 0.5%)
Major complications	6956	116 (1.7 ± 2.3%)
Neurologic complications		
Stroke	3695	42 (1.1 ± 2.4%)
Paraplegia	1106	4 (0.4 ± 0.0%)
Stent graft induced new entry	6532	32 (0.5 ± 0.0%)
Retrograde type A AD	6532	23 (0.4 ± 0.0%)
Distal AD	6532	9 (0.1 ± 0.0%)
Surgical conversion	8885	25 (0.3 ± 0.0%)
Adjunctive endovascular procedures	8885	25 (0.3 ± 0.0%)
Mortality	8316	137 (1.6 ± 0.9%)
Procedure related	7754	65 (0.8 ± 0.0%)
Non-procedure related	7754	48 (0.6 ± 0.0%)

AD: aortic dissection.

### Follow-up data

**Figure 5:**
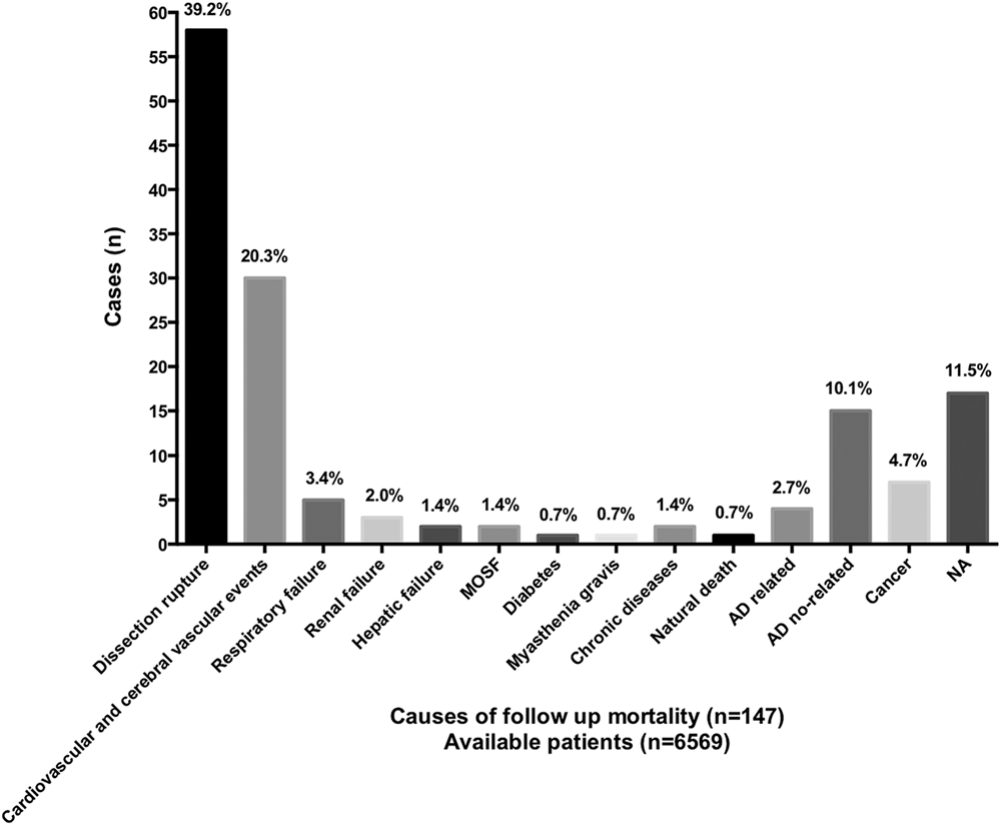
Types and proportions of long-term death causes. AD: aortic dissection, MOSF: multiple organ system failure, NA: not applicable.

**Figure 6:**
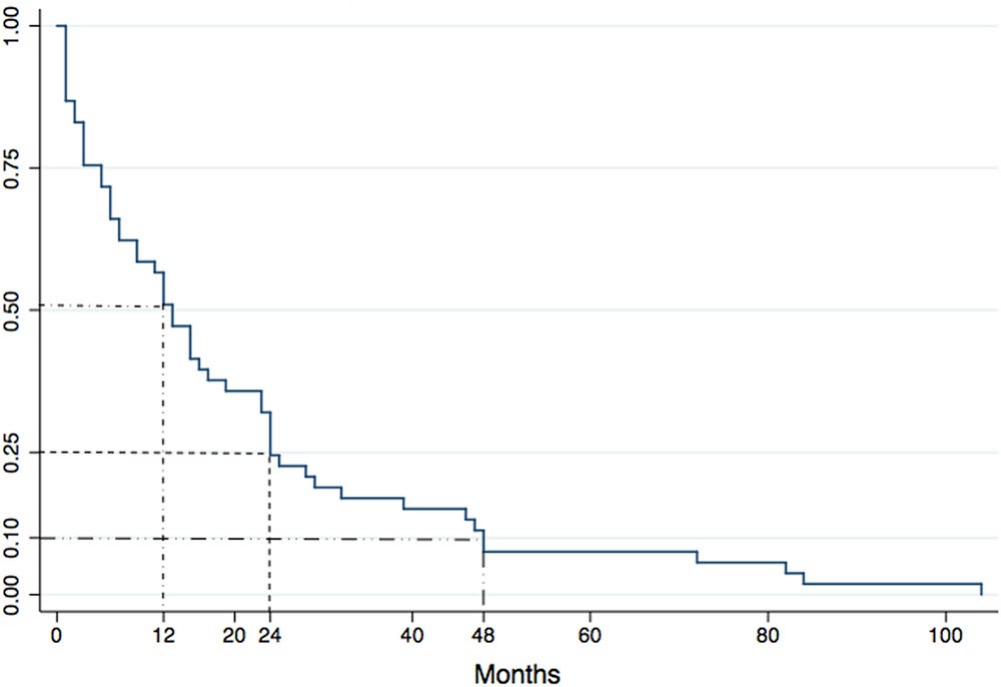
Kaplan-Meier survival curve for long-term death. Death at one- and two- year follow-up accounted for about 50% (short dash-dot line) and 75% (dot line) of long-term deaths, respectively. 90% (long dash-dot-dot line) of deaths occurred within 4 years follow-up.

Patients were examined after one month from discharge using computed tomographic scanning. Major follow-up parameters are summarized in Table 4. Follow-up information was available for 8387 patients. Time to follow-up (1–132 months) was available for 6883 patients. Late major complications were reported in 1.6% of 5942 cases. The most critical long-term complications were related to retrograde type A AD (0.3% of 6192 cases) and stent-graft related distal AD (0.5% of 6192 cases). Among 7938 patients with available data, late surgical conversions were performed in 0.2% cases, and adjunctive endovascular stent-graft procedures were performed in 1.1% cases. Type and proportion of long-term death causes are illustrated in Fig. 5. Dissection rupture accounted for most death (39.2%). Among the 17 dissection rupture patients with available information on death timing, 35.3% patients and 58.8% patients died within 6 and 12 months after discharge, respectively. Figure 6 shows the Kaplan-Meier survival cure for long-term death among patients with available information. Death rate at one- and two- year follow up was about 50% and 75%, respectively; and 90% within 4 years of discharge. Figure S4 depicts procedure- and nonprocedure-related deaths during follow up, which were statistically similar (1.0% vs. 1.3%, P = 0.737).

**Table 4:** Follow up data.

	Data available (n)	No. of events [n(% ± SE)]
Follow up patients	8885	8387 (94.4%)
Duration of follow up (month)	6883	1–132
Major complications	5942	97 (1.6%)
Stent graft induced new entry	6192	53 (0.8 ± 0.0%)
Retrograde type A AD^*^	6192	21 (0.3 ± 0.0%)
distal AD	6192	32 (0.5 ± 0.0%)
Surgical conversion	7938	19 (0.2 ± 0.0%)
Adjunctive endovascular procedures	7938	87 (1.1 ± 0.0%)
Mortality	7001	164 (2.3 ± 1.1%)
Procedure related	6569	64 (1.0 ± 0.0%)
Non-procedure related	6569	83 (1.3 ± 0.0%)

^*^AD: aortic dissection.

## Discussion

The main findings in the present meta-analysis of 2008–2015 publications from mainland China on TEVAR for type B AD are a change in the geographic and specialty practice of TEVAR and apparently improved safety outcomes relative to prior time periods in mainland China and Western countries.

The distribution of medical resources and development of medical technique is unbalanced across different regions of mainland China. Beijing and Shanghai with the most advanced medical resources and techniques have attracted a large proportion of patients living in other regions of China. However, in 2008–2015, the difference in reported TEVAR numbers between Beijing/Shanghai and other locations decreased as compared to that of in 2001–2007^2^, suggesting a rapidly increasing use of TEVAR during the past eight years in other regions of China, with the exception of no reports from Tibet, by 9- and 4-fold in the number of TEVAR and related studies, respectively.

In the past eight years, the shift in specialist type treating type B AD in mainland China from interventional radiologists to cardiologists and cardiothoracic surgeons while stable for vascular surgeons may reflect that more cardiologists and cardiothoracic surgeons are now capable of performing TEVAR; moreover, because interventional radiology is usually not the first specialty to which type B AD patients are referred, cardiologists, and cardiothoracic and vascular surgeons might become the main specialists treating type B AD in China.

The mean age of 53.9 years for type B AD patients in China is 7–9 years younger than those reported by Eggebrecht’s^3^, Hiratzka’s^10^ and Patel’s^11^ studies, suggesting an earlier onset of type B AD among Chinese compared with Caucasians in Europe and North America. Although published data suggest a lower prevalence of atherosclerosis among Chinese AD patients as compared to Caucasians, rates of postoperative neurological complications, namely stroke (p = 0.899) and paraplegia (p = 0.319) were statistically similar in this and the study by Eggbrecht *et al*.^3^ (Table 5). When compared with the study by Moulakakis *et al*. on a similar data collection time period (2006–2013), rates of stroke (p = 0.722) and paraplegia (p = 0.049) were statistically similar to those in our study for acute uncomplicated type B AD cases in the study by Moulakakis *et al*.^12^. However, stroke (p = 0.001) and paraplegia (p = 0.007) rates were lower in our study as compared to the data from acute complicated type B AD in the study by Moulakakis *et al*.^12^ (Table 6).

**Table 5:** Results of comparison among the study by Eggbrecht *et al*., Chinese data (2001–2007) and Chinese data (2008–2015).

	Eggebrecht (1999–2004)		China (2001–2007)		China (2008–2015)		P value	
	Data available n[%]		Data available (n[%])		Data available (n[%])		China (2008–2015) vs. China (2001–2007)	China (2008–2015) vs. Eggebrecht (1999–2004)
Number of publications		38		39		152	—	—
Patients/center (median)		10(3–127)		16(3–179)		30(5–578)	—	—
Procedure success	551(90.5%)	98.2 ± 0.5%	1301(99.8%)	99.2 ± 0.8%	8885(74.8%)	99.1 ± 0.8%	0.203	0.076
Major complications	449(73.7%)	11.2 ± 1.4%	1284(98.5%)	3.4 ± 0.1%	6956(58.6%)	1.7 ± 2.3%	<0.001	<0.001
Stroke	518(85.1%)	1.9 ± 0.6%	1284(98.5%)	0.2 ± 0.0%	3695(31.1%)	1.1 ± 2.4%	<0.001	0.899
Paraplegia	609(100%)	0.8 ± 0.4%	1284(98.5%)	0	1106(9.3%)	0.4 ± 0.0%	0.003	0.319
In-hospital mortality	524(86.0%)	5.3 ± 0.9%	1304(100%)	2.6 ± 0.1%	8316(70.0%)	1.6 ± 0.9%	<0.001	0.001
In-hospital retrograde type A dissection	429(70.4%)	1.9 ± 0.6%	1301(99.8%)	0.4 ± 0.0%	6532(55.0%)	0.4 ± 0.0%	0.099	<0.001
Long term mortality (Follow up month)	609(100%)(19.5 ± 7.1)	4.1 ± 0.9%	1033(79.2%)(27.1 ± 17.5)	1.5 ± 0.1%	7001(58.9%)(1–132)	2.3 ± 1.1%	0.41	<0.001

**Table 6:** Results of comparison among the study by Moulakakis *et al*. (2006–2013) and Chinese data (2008–2015).

	Moulakakis (2006–2013)		Moulakakis (2006–2013)		China (2008–2015)		P value	
	Data available (n[%]) Acute complicate type B AD		Data available (n[%]) Acute uncomplicated type B AD		Data available (n[%])		China (2008–2015) vs. Moulakakis (2006–2013) acute complicated AD	China (2008–2015) vs. Moulakakis (2006–2013) acute uncomplicated AD
Number of publications	30		15		152			
Stroke	2246(88.7%)	3.9 ± 3.8%	1520(64.8%)	1.6 ± 4.6%	3695(31.1%)	1.1 ± 2.4%	0.001	0.722
Paraplegia	2334(92.2%)	3.0 ± 4.2%	1654(70.5%)	0.8 ± 1.5%	1106(9.3%)	0.4 ± 0.0%	0.007	0.049
In-hospital mortality	2531(100%)	8.5 ± 7.0%	2347(100%)	4.8 ± 3.4%	8316(70.0%)	1.6 ± 0.9%	<0.001	0.041
Long term mortality (Follow up month)	807(31.9%)(34.9 ± 14.5)	23.4 ± 9.7%	2101(89.5%)(44.0 ± 21.8)	20.4 ± 14.4%	7001(58.9%)(1–132)	2.3 ± 1.1%	<0.001	<0.001

While the results of the present study indicate that TEVAR procedural success rate in China has remained stable over the past 15 years and comparable to that in Western countries, in-hospital mortality appeared to improve in mainland China from 2001–2007^2^ to 2008–2015, becoming significantly lower to that in the study by Eggebrecht *et al*.^3^. A similar trend was found when data from the present study were compared to those from the study by Moulakakis *et al*. (in acute complicated type B AD [p < 0.001] and in acute uncomplicated type B AD [p = 0.041]) (Table 6), and to the high in-hospital mortality (30% in complicated patients aged older than 70 years, and 10.1% in those younger than 70 years) from the International Registry of Acute Aortic Dissection (IRAD) study^11^. The latter differences might be attributed to: continuous indication expansion and technical improvement for TEVAR during the past 8 years^6,13^; and younger age of patients undergoing TEVAR in China than in Western countries, with reduced risk for postoperative cardiovascular and cerebrovascular adverse events or death.

Incidence of retrograde type A AD after TEVAR was significantly lower (approximately one fifth) both in 2008–2015 and 2001–2007 Chinese studies than in the study by Eggbrecht *et al*.^3^, despite: (1) respective proportion of acute cases of 70%, 36% and 58.1%, which have been associated with a higher proportion of stent-graft induced new intimomedial tear due to very fragile aortic wall in acute stage of AD^14–17^; and (2) less conservative preference by Chinese surgeons during the past 8 years for 15% oversizing of stent-grafts. The lower incidence of post TEVAR retrograde type A AD tear might be explained by younger mean age in the Chinese patient population with less atherosclerotic burden in the landing zone allowing stronger resistance to stent-graft radial pressure, and increased use of domestic stent-grafts (over half of stent-grafts used in 2001–2007) with more recently optimized curve shaped design to better adapt to the anatomy of arch-descending aorta anatomy^8,18^, and stent-graft struts designed to be in compliance with the upward convexity and forward convexity of arch-descending aorta to reduce intima trauma^19–21^.

Improvement in TEVAR diagnosis and timely intervention during the last 8 years might have been an important determinant in reducing nonprocedure-related mortality during hospitalization. Because of in-hospital death predominantly (75%) occurring during the first week after TEVAR and secondary to AD rupture, more attention should be paid during this period to signs and symptoms of impending AD rupture, such as persistent and aggressive chest pain, refractory hypertension, pleural effusion and proximal type I endoleak^22^, in which case aorta CTA should be performed as soon as possible to ensure timely re-intervention.

Lower follow-up mortality in 2001–2007 and 2008–2015 Chinese data relative to that in the study by Eggebrecht *et al*.^3^, and lower follow-up mortality in 2008–2015 Chinese data relative to that in the study by Moulakakis *et al*.^12^ and the rather high 5-year mortality (16%) in the IRAD study^11^, also might be attributed to the younger Chinese population with less comorbidity. Importance of surveillance extends to follow-up mortality with 33% and 60% of deaths occurring within the first 6 and 12 months of follow-up, respectively and 90% up to 4 years follow-up; therefore, continuous monitoring and disease management are most important and great attention should be paid to changes in AD morphology and signs of rapid growth of dissection and impending rupture (intermittent dull chest pain^23^ or hoarseness of the voice^24^) to ensure timely reintervention.

Although the present study provides a contemporary overview of use and effectiveness of TEVAR in treatment of type B AD in mainland China, including data by province, autonomous region and municipality, it has several limitations. Overall, the generalizability of the results of the present meta-analysis on prevalence of type-B dissection and major complication rates after TEVAR is limited by the high proportion of studies included that did not report pertinent data because of their research focus and design; information on several variables therefore was available for about 50% and in some cases less of total patients in the meta-analysis. More robust meta-analyses are warranted including future studies providing more comprehensive datasets. First, many studies did not clarify if type B AD was complicated or uncomplicated, which might influence post-TEVAR complication and mortality rate estimation thereby leading to misclassification bias when comparing to other meta-analyses. Second, most studies did not report stent-graft type, rendering unfeasible to accurately determine the effect of various types on TEVAR outcomes, especially endoleak and stent-graft-induced new entry. Third, because doctors from medical school affiliated, teaching and tertiary hospitals usually are more motivated to publish than those from non-tertiary hospitals; generalizability of study results might be limited. Fourth, beyond traditional hard endpoints, i.e., death and major complications, false lumen remodeling has been proposed as an important long-term outcome post TEVAR; however, few studies have reported on it. Fifth, most studies did not report patient comorbidities, such as cardiovascular and cerebrovascular diseases, which would bias postoperative morbidity and mortality estimation. Sixth, single center series included in this study might include highly selected patients that may introduce publication bias. Seventh, because follow up time in studies in the 2008–2015 Chinese dataset was expressed as a range of time and not a particular timepoint, overall median follow-up duration could not be calculated for comparisons with other published meta-analyses. Finally, the use of adjunctive techniques (such as debranch and chimney) in TEVAR has increased during the past 8 years, which may also increase the risk of procedure-related and postoperative morbidity; however, scarce information on their use did not allow to control for the related confounding effect.

In conclusion, the present study suggests that use and effectiveness of TEVAR for type B AD treatment in mainland China has improved over the years, achieving short- and long- term outcomes that are comparable and some possibly more favorable than those in Western countries. Further effort is required to establish more reliable and informative reporting protocols and systems at a national level to ensure generation of more comprehensive and generalizable evidence to guide future medical development in China.

## Electronic supplementary material

supplemental information
