# Supplementary material for: Recent evolution in use and effectiveness in mainland China of thoracic endovascular aortic repair of type B aortic dissection
Source: Sci Rep. 2017 Dec 11;7:17350. doi: 10.1038/s41598-017-17431-w (PMC5725573; doi:10.1038/s41598-017-17431-w)
Supplement: Supplementary file 1 — supplemental information [file 41598_2017_17431_MOESM1_ESM.doc]

**Recent evolution in use and effectiveness in mainland China of thoracic endovascular aortic repair of type B aortic dissection**

Jiang Xiong, MD, PhD^1^*, Chen Chen, DrPH^2^, Zhongyin Wu, MD^3^, Duanduan Chen^4^, PhD, Wei Guo, MD^1^*

From the ^1^Department of Vascular and Endovascular Surgery, Chinese PLA General Hospital, Beijing, P.R. China; ^2^Department of Health Policy and Management, Jiann-Ping Hsu College of Public Health, Georgia Southern University, Statesboro, GA, USA; ^3^Department of Vascular Surgery, Affiliated Hospital of Chengde Medical College, Chengde, Hebei, P.R. China; ^4^Department of Biomedical Engineering, School of Life Science, Beijing Institute of Technology, Beijing, P.R China

Jiang Xiong and Chen Chen are co-first authors

*Correspondence author: Jiang Xiong & Wei Guo, Department of Vascular and Endovascular Surgery, Chinese PLA General Hospital, 28 Fuxing Rd, Haidian District, Beijing, 100853, China;

Tel: 86-10-66938349; Fax: 86-10-68176994; E-mail:xiongjiangdoc@126.com

Table S1. List of variables/ information extracted from selected articles

| **List of variables abstracted from each article (if available)** | |
| --- | --- |
| **Study characteristics** | **Procedural data** |
| No. of hospitals involved | Types of anesthesia |
| No. of departments involved | Procedure success |
| Geography | Covered left subclavian artery procedure |
| Specialist types involved | Debranch procedure |
|  | Chimney procedure |
| **Patient characteristics** |  |
| No. of patients with type B AD | **Follow-up data** |
| Age | Duration of follow-up |
| Sex | Late major complications |
| Acute AD | Late retrograde type A AD |
| Hypertension | Late stent graft related distal AD |
| Diabetes | Late surgical conversion |
|  | Adjunctive endovascular procedure during follow-up |
| **In-hospital data** | Late mortality |
| Major complications | Late causes of mortality |
| Paraplegia | Late procedure related mortality |
| Retrograde type A AD | Late non-procedure related mortality |
| Stent graft related distal AD |  |
| Early surgical conversion |  |
| Adjunctive endovascular procedure |  |
| In-hospital mortality |  |
| Causes of mortality |  |
| Procedure related mortality |  |
| Non-procedure related mortality |  |


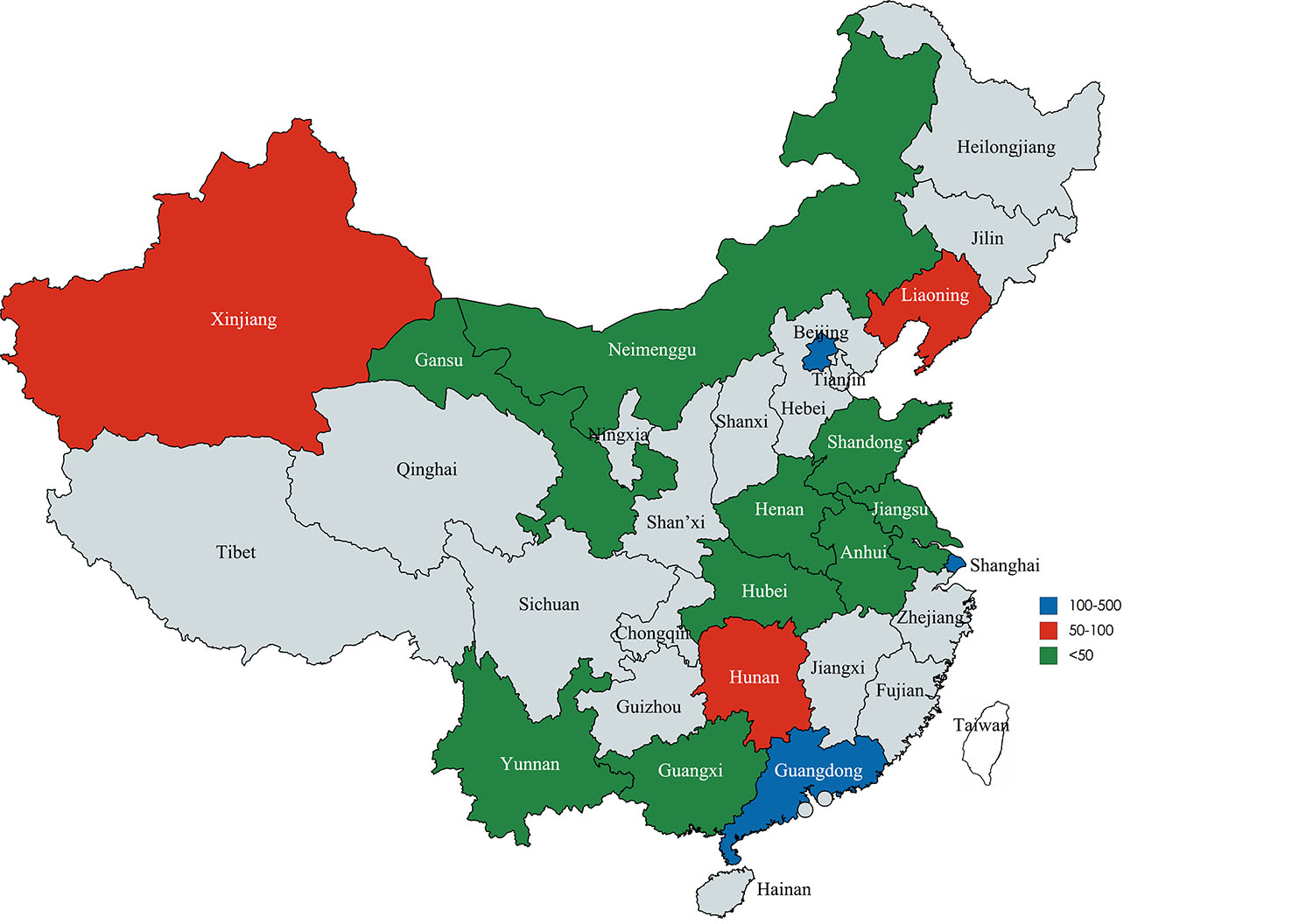


Figure S1. Geographical distribution of TEVAR cases in 16 administrative regions of mainland China (Jan. 2000-Dec. 2007). The number of cases was categorized into 3 levels (green: <50; brown: 50-<10;, and blue: 100-500). This map is created using the “mapchart” online figure generator (https://mapchart.net/).


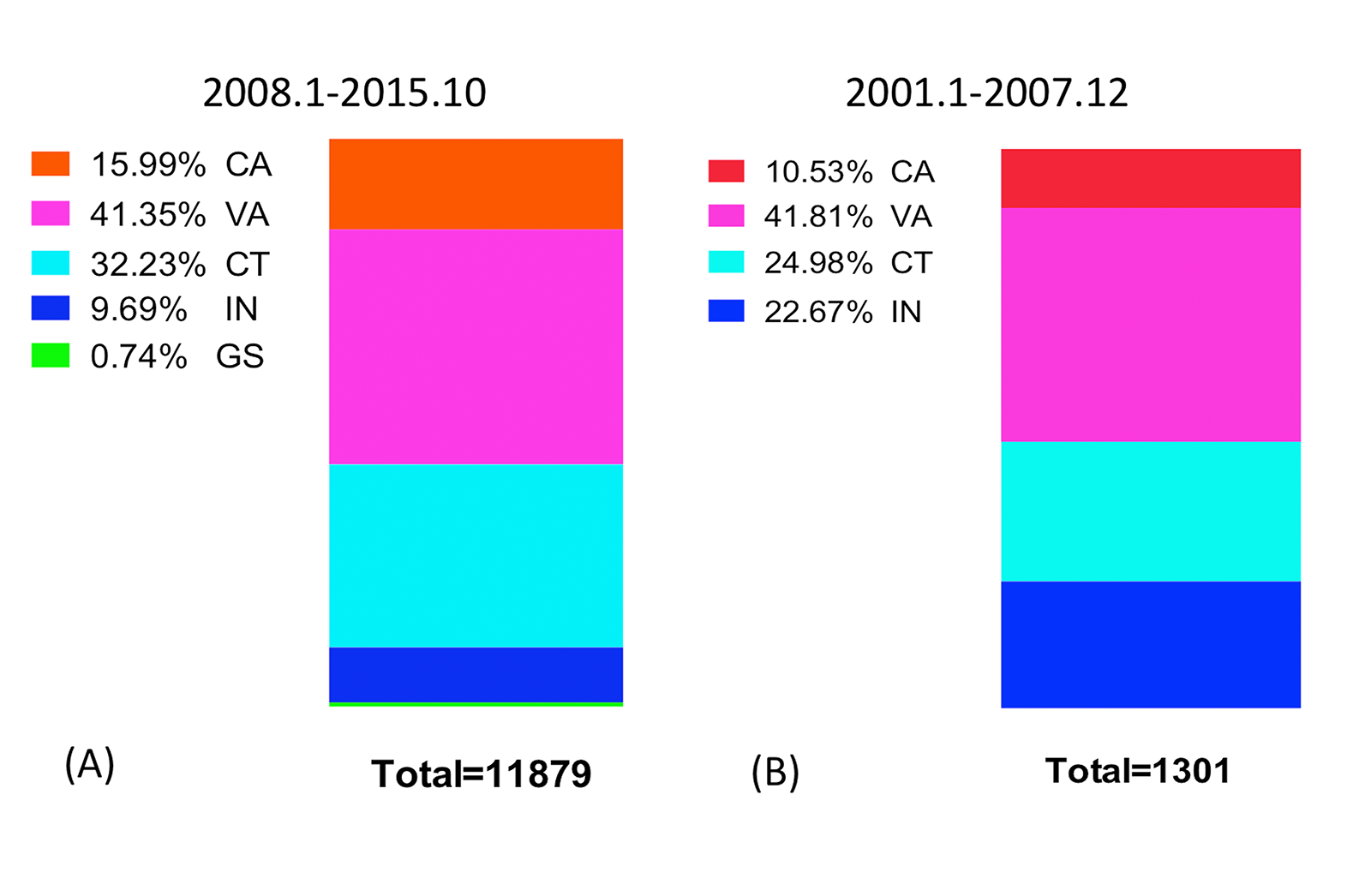


Figure S2. Number of TEVAR procedures performed by different specialists.

A: Chinese data (Jan. 2008-Oct. 2015). B: Chinese data (Jan. 2000-Dec. 2007). CA: cardiology, VA: vascular surgery, CT: cardiothoracic surgery, IN: interventional radiology, GS: general surgery.


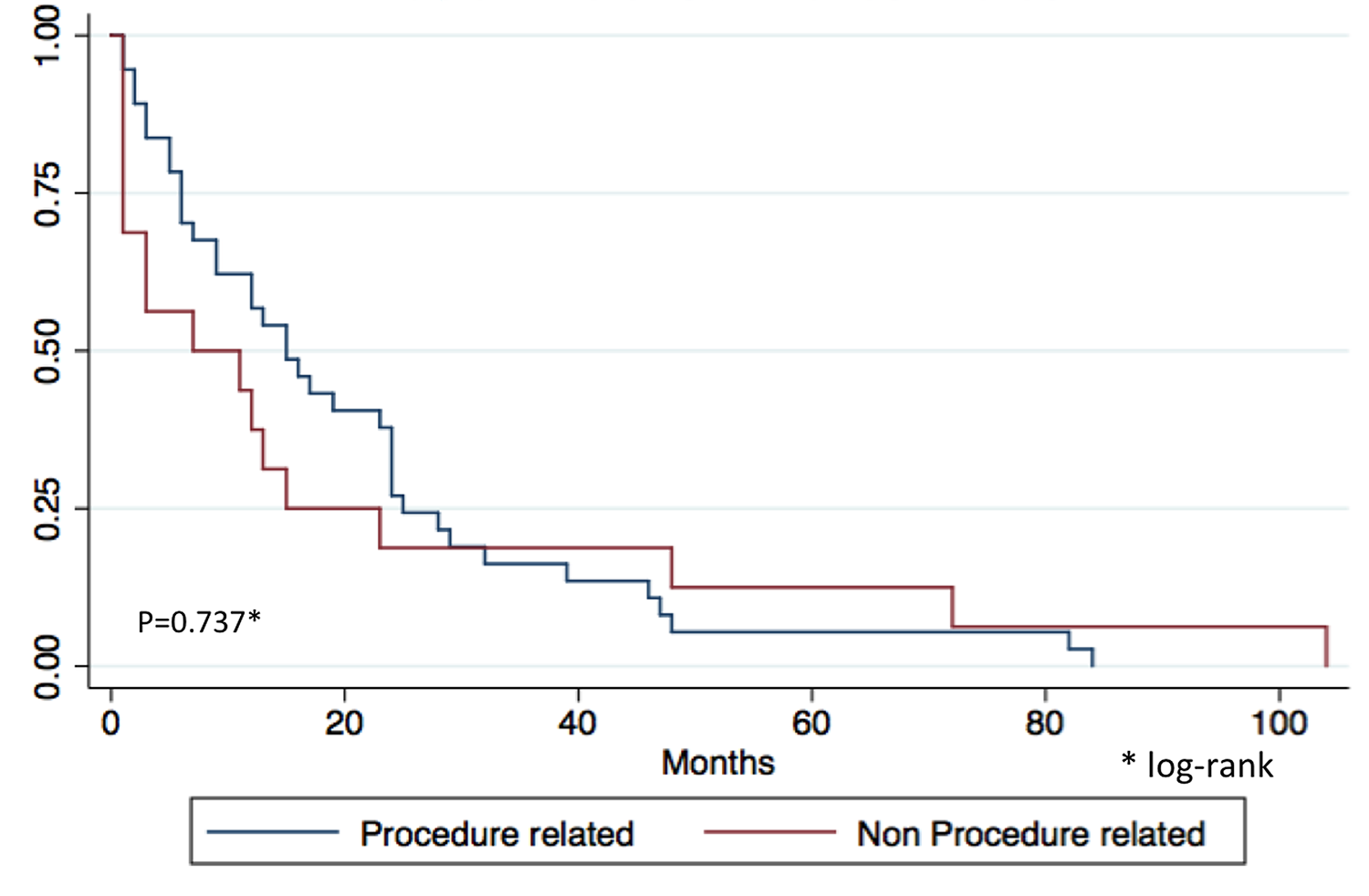


Figure S3. Thirty-day mortality among patients who underwent TEVAR with procedure-related adverse events relative to those with non-procedure-related adverse events. The death curves were generated by using the Kaplan–Meier method. There was no significant difference between non–procedure-related (red line) and procedure-related (blue line; 0.8±0.0% vs 0.6±0.0%, log-rank P =0.765) mortality.


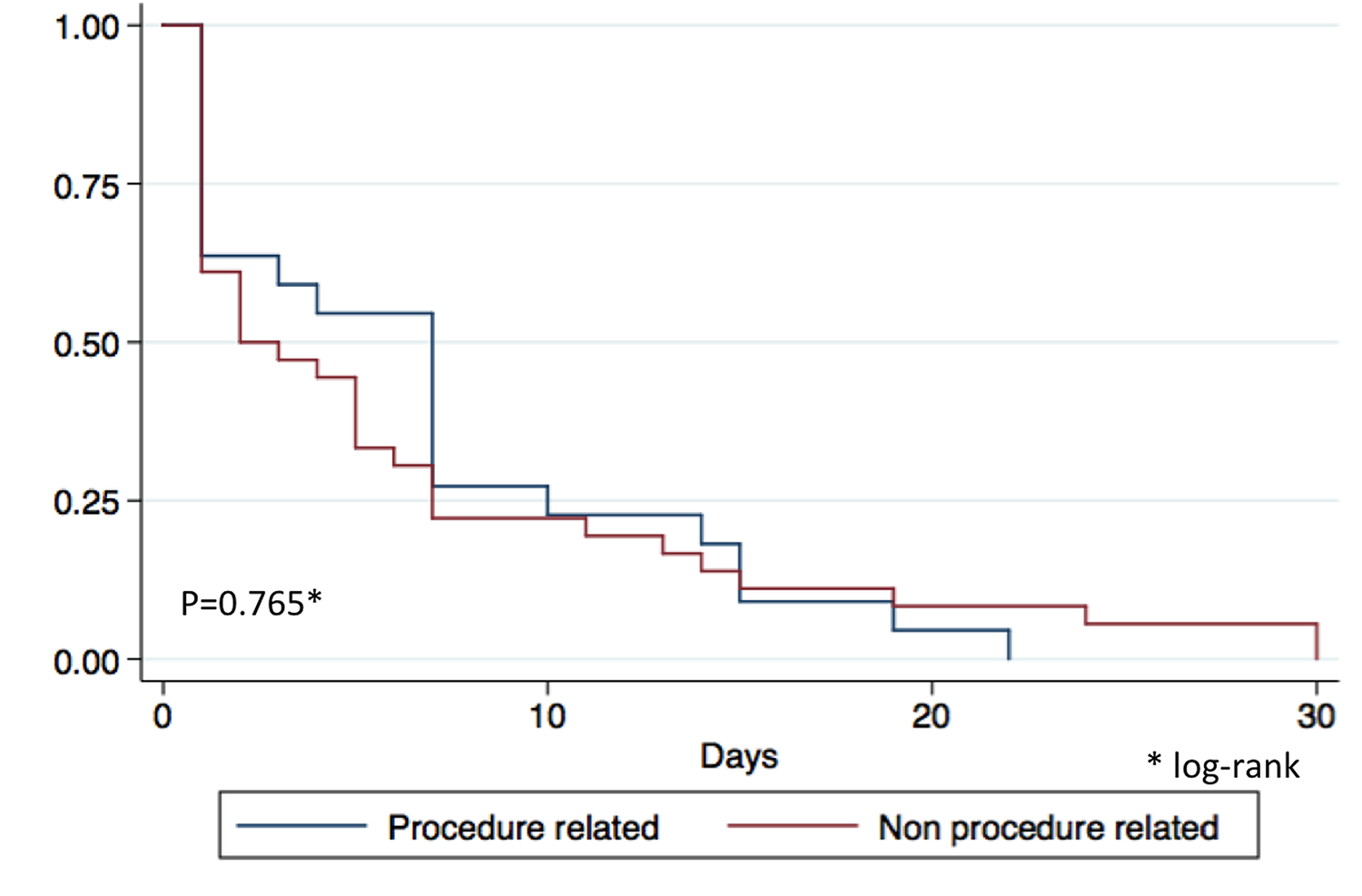


Figure S4. Long-term mortality among patients who underwent TEVAR with procedure-related adverse events relative to those with non-procedure-related adverse events. The death curves were generated by using the Kaplan–Meier method. There was no significant difference between non–procedure-related (red line) and procedure-related (blue line; 1.0±0.0% vs 1.3±0.0%, log-rank P =0.737) mortality.

1. Cao Z, Yu C, Meng G. Study on the effects of antigen retrieval on HER-2 detection in gastric cancer. *Acta Univ Med Anhui*. 2013,48(9):1127-1129.

2. Yu H, Ma Y, Yuan Q, Ma X. Application of the restrictive stent for prevent and treat of the distal redissection after endovascular repair for Stanford type B aortic dissection. *J Xinjiang Med Univ*. 2013,36(9);1330-1333.

3. Zhang C, Wu J, Liu H, Zhao J, Li X, et al. Analysis of endovascular repair for Stanford B aortic dissection. *MMJC*.2013,15(8):21-22.

4. Li F, Wei C, Cao J. Application effect of endovascular stent exclusion for descending aortic aneurysm. *Jilin Med J*. 2013,43(21):4323-4324.

5. Zhan B, Liu J, Du S, Zhang F, Chen H. Endovascular stent-graft Exclusion for Standford B Aortic Dissection. *Chin J Clin Thorac Cardiovasc Surg*.2013,20(3):360-361.

6. Hui H. Nursing care of 67 patients with Stanford B aortic dissection. *Inner Mongol JTCM*. 2013.32(16):178.

7. Shen G, Yang J, Chen J, Sun C, Wang D, et al. Efficacy of stent graft exclusion for Stanford B aortic dissection in critically ill patients. *J Clin Med Pract*. 2013,17(11):68-69.

8. Bin T, Li Y, Da G, Wu J, Li Z, et al. Endovascular graft exclusion of Stanford B thoracic aortic dissection aneurysm in 12 patients. *Qinghai Med J*. 2013,43(3):11-12.

9. Li Q, Jiang Y. Coordination and nursing care of endovascular stent graft repair for Standford B aortic dissection. *Jiangsu Med J*. 2013,39(4):491-492.

10. Liu J, He Y, Zhang Y, Zhou H, Ma R. Clinical Analysis of Stent Endovascular Graft Exclusion for Stanford Type B Aortic Dissection. *Guede Chin Med*. 2013,11(5):19-21.

11. Yang H, Fan B, Huang J. To discuss the nursing in endovascular graft exclusion for Stanford B aortic dissection. *Today Nurs*. 2013:17-19.

12. Li Y, Li J. Clinical Analysis of Endovascular Graft Exclusion for Stanford Type B Aortic Dissection Aneurysm. *J Inner Mongolia Med Univ*. 2013,35(s1):49-52.

13. Li B, Ju X, Cheng Y, Wang Y. Nursing care of endovascular repair for Stanford B aortic dissection aneurysm in 16 cases. *Chin J Misdiagn*. 2011,11(14):3449.

14. Li G, Zhang Y. Analysis of 45 Cases of Stanford B Aortic Dissection Aneurysm after Endovascular Graft Exclusion in Xining Area. *Qinghai Med J*. 2013,43(1):4-6.

15. Wang Y, Li Y, Zhong H. Clinical Analysis of 32 cases of Stanford B Aortic Dissection. *Pub Med Forum Mag*. 2013,17(1):34-35.

16. He Y, Zhao X, Jin J. Endovascular graft exclusion in patients with aortic dissection stanford type B:early and mid-term results. *J Clin Cardiol*. 2012,28(12):925-928.

17. Xu L, Sun Y, Yuan H, Kong X, Zhang S, et al. Misdiagnosed Analysis and Therapeutic Summary of 8 Patients with Painless Stanford B Aortic Dissection. *Clin Misdiagn Misther*. 2012,25(11):9-11.

18. Lu T, Wang X, Mao C. Observation and Nursing of Stanford B Aortic Dissection Stent Intervention Surgery. *Clin Med*. 2012,19(11):1990-1991.

19. Wang Y. Observation and nursing care of endovascular graft exclusion for the treatment of Stanford B aortic dissection aneurysm. *J Nurs Training.* 2012,27(20):1858-1860.

20. Jiao F. Application of evidence based nursing in stent graft for treatment of aortic dissection. *J Qilu Nurs*. 2012,18(29):98-99.

21. Zhou J, Yuan Z, Huang J, Re N, Shan W. Report of 19 cases of endovascular graft exclusion of Stanford type B aortic dissection by stent graft implantation. *J Clin Exp Med.* 2012,11(19):1529-1530.

22. Ge X, Fang Q, Guan S, Sai L, Ren H. Thoracic Endovascular Aortic Repair for Type B Aortic Dissection (134 cases). *Chin J Min Inv Surg*. 2012,12(9):774-776.

23. Ding G, Chen S, Dong C, Shi Y, Shao A. Stent grafting for thoracic aortic diseases. *Chin Heart J*. 2012,24(5):642-645.

24. Gu Y, Guo L, Qi L, Yu H, Li X, et al. Report of 98 cases of Type B aortic dissection. *Chin J Min Inv Sur*. 2012,12(8):675-677 686.

25. Gao Y, Zhang C. Report of endovascular stent implantation for acute Stanford B aortic dissection aneurysm in 8 cases. *J Aerospace Med*. 2012,23(7):856-857.

26. Xiao Z, Ma J, Zhai Y, He Q, Jin L. Clinical analysis of endovascular stent implantation for Stanford B aortic dissection in 36 cases. *Chin J Clin Res*. 2012,25(6):568-569.

27. Kan J, Chen S, Zhu J, Lin L, Xu H, et al. Clinical application of rotational 3D-DSA in "One-stop" operation for aortic dissection with coronary artery disease. *Mod Instruments*. 2012,18(3):51-54.

28. Mu H. Perioperative nursing care of B type Stanford aortic dissection treated with endovascular stent graft. *Tianjin J Nurs*. 2012,20(2):82-83.

29. Bao H, Zheng X. Perioperative nursing care of endovascular graft exclusion for Stanford B aortic dissection. *Anhui Med J*. 2012,33(4):496-497.

30. Liu H, Dai X, Luo Y, Wei M. Thoracic Endovascular Aortic Repairing Treatment with Stent-Graft for Stanford Type B Aortic Dissection. *Chin J Surg Integr Tradit West Med*. 2012,18(2):148-150.

31. Haerman A, Reyihanguli M, Sailike M, Luo J, Aizezi A, et al. Endovascular stent graft repair for the treatment of B Stanford aortic dissection aneurysm: a report of 72 cases. *Chin J Pract Surg*. 2012,32(3):240.

32. Cao K, Ge Y, Cuan G, Zhu L, Li Y, et al. Analysis of endovascular treatment of type B aortic dissection. *Contempor Med*. 2012.18(6):11-12.

33. Wang G, Zhai S, Li T, Shi S, Zhang K, et al. Complications after thoracic endovascular aneurysm repair for Standford B dissection. *Chin J Interv Imaging Ther*. 2012,9(2):87-90.

34. Shi T, Xie X. Early and Midterm Follow-Up Results of Endovascular Repair of Stanford Type B Aortic Dissection：Report of 85 Cases. *Chin J Bases Clin General surg*. 2012,19(1):66-71.

35. Yang D, Li G. Clinical analysis for the treament of Stanford B aortic dissection with interventional physicians in Department of Cardiology. *Chin Foreign Med Treat*. 2012,31(2):87.

36. Chen W, Yu J, Yang R, Bai L, Li Y, et al. Therapy of Stanford B aortic dissecting aneurysm using China － made tectorial stent. *Med J NDFNC*. 2011,32(6):434-436.

37. Liu X, Liu J, Zhou X, Yu B, Wu Q, et al. Endovascular stent-graft Exclusion for Standford B Aortic Dissection Aneurysm. *Chin J Clin Med*. 2011,18(6):777-779.

38. Zhang L, Ding S, Zhang J, Yang Y, Ouyang H, et al. Clinical observation of endovascular graft exclusion on 30 cases of Stanford type B dissecting aortic aneurysm. *Med J NDFSC*. 2011,21(12):1318-1320.

39. Li Z, Ni C, Jin Y, Shen Z, Huang H, et al. Endovascular stent graft repair for Standford B Aortic Dissection. *Suzhou Univ J Med Sci*. 2011,31(6):981-983.

40. Wang H. To investigate the nursing experience of perioperative of endovascular repair in Standford B acute aortic dissection patients. *Contempor Med*. 2011,17(28):129-130.

41. Hou P, Li Q, Su Y. A retrospective analysis of endovascular repair for Stanford B aortic dissection in 25 cases. *J Qiqihar Med Coll*. 2011,32(15):2417-2419.

42. Zheng H, Wang X, Wang R, Hu X, Qian Y. Clinical application of endovascular graft exclusion in the treatment of Stanford B aortic dissection. *Chin Foreign Med Res*. 2011,9(20):34-36.

43. Bai T, Deng S. Xiong R, Liu Y. Endovascular graft exclusion combined hybridization in the treatment of aortic dissecting aneurysm. *Jilin Med J*. 2011,32(17):3482-3484.

44. Tan D, Tan W, Yang K, Liao K, Zhang W. The endovascular repair of Stanford type B aortic dissection: early and midterm results of 32 cases. *Chongqing Med.* 2011,40(12):1167-1168 1171.

45. Zheng H, Liu J, Xu L, Zhong J. Diagnostic Value of MSCTA for Stanford B Aortic Dissection Prior to Stent-Graft Placement. *Chin J Med Imagine*. 2011,19(4):244-247.

46. Qiao J, Zhang X, Guo Z. Treatment options for type B aortic dissection or bow reduction. *Inner Mongolia Med J*. 2011,43(3):340-341.

47. Liu X, Li Y, Niu T, Chen J, Zhang Y, et al. Endovascular graft exclusion for the treatment of Stanford B aortic aneurysm. *J Ningxia Med Univ*. 2011,33(3):291-292

48. Tian S, Li Y, Chen J. Application of Roy adaptation model in nursing care of patients with aortic dissection. *J Pract Med*. 2011,27(6):1093-1094.

49. He Q, Pang Z, Yuan P, Zhang Y, Ye H. Application of three dimension navigation technology in endovascular stent graft exclusion in the treatment of Stanford type B aortic dissection. *Chin J Interv Imaging Ther*. 2011,8(2):98-100.

50. Sun Z, Tian Z, Liu P, Liu T. Endovascular graft exclusion of Stanford B thoracic aortic dissection in 21 patients. *J Chin Pract Diagn Ther*. 2011,25(3):268-269.

51. Jia J, Zu Z, Yin X, Qin Y, Jiang Y. Nursing experience of endovascular stent graft exclusion combined with artificial blood vessel bypass surgery for type aortic dissection in 5 cases. *China Pract Med.* 2011,6(7):211-212.

52. Wang X, Jin Q, Han Y, Liu X, Jin H, et al. Endovascular graft exclusion of Stanford B aortic dissection in 210 patients. *Chin J Pract Intern Med*. 2011,31(1):45-47.

53. Jiang H, Li B, Chen Y, Wang Y, Wei Y. Endovascular repair for the treatment of Stanford B aortic dissection. *J HBUM*. 2010,29(6):525-526.

54. Wang P, Shi J, Zhao X. Preoperative and postoperative nursing care of endovascular graft exclusion for Stanford B aortic dissection. *J Nongken Med*. 2010,32(6):559-560.

55. Zhou Z, Huang J, Wu W, Chu W, Qiu Z, et al. Clinical Analysis of Endovascular Graft Exclusion for the Treatment of Stanford Type B Aortic Dissection. *Chin J Gen Pract*. 2010,8(12):1516-1518.

56. Yang Q, Qiu F, Shan Z, Liao C, Zhu P. Evaluation of endovascular graft exclusion in treatment of aortic dissection of Stanford B. *J Clin Cardiol*. 2010,26(11):821-823.

57. Cai H, Zhang C, Jin H. Endovascular Aortic Repairing Treatment for Stanford Type B Thoracic Aortic Dissection. *J Kunming Med Univ*. 2010,(10):64-67.

58. Tian H. Vascular stent placement in treatment of thoracic aortic dissection Stanford B 33 cases. *J Med Forum*. 2010,31(8):51-52.

59. Qiao T, Liu C, Liu C, Huang D, Wang W, et al. Endovascular treatment and mid term efficiency of type B aortic dissection. *Chin J Vasc Surg*. 2010,2(3):148-151.

60. Wang H, Zheng Y, Wang J, Cheng J, Shi F, et al. Thoracic endovascular aortic repair of Stanford B type aortic dissection. *Med J NDFNC*. 2010,31(4):282-284.

61. Zhong Z, Li B, Li C. Clinical study on the treatment of type B aortic dissection in 25 cases with covered stent. *Chin J Vasc Surg*. 2010,2(2):121-123.

62. Song B, Liu R, Liu D, Tang H, Zhang B, et al. Endovascular repair of Stanford B aortic dissection A report of 16 patients. *J Fourth Mil Med Univ*. 2009,30(24):3129-3131.

63. Yu J, Huang L, Jiang S, Jin J, Lv B, et al. Complications following transluminal stent-graft placement for aortic dissections. *Contemporary Med*. 2009,15(29):532-535.

64. Liu X, Sun M, Lu M, Huang X, Lu X, et al. Demonstration of the Adamkiewicz artery and its application in the endovascular therapy of thoraco abdominal aortic disease. *J Surg Concepts Pract*. 2009,14(3):297-301.

65. Meng W, Zhang E, Yang J, Guo Y, An Q, et al. Surgical Treatment of Aortic Dissection. *Chin J Chin Thorac Cardiovasc Surg*. 2009,16(1):40-42.

66. Lin X. Analysis of 20 cases of aortic dissection complicated with acute coronary syndrome. *Chin J Misdiagn*. 2009,9(4):954-955.

67. Zhu Z, Chen X, Luo C, Tan Y, Xu G, et al. Influence of stent-graft endovascular exclusion therapy on blood flow in Debakey B aortic dissection. *J Clin Rehabilit Tiss Eng Res*. 2008,12(52):10263-10267.

68. Yuan J, Lin Y, Liu L. Experience of endovascular exclusion for Stanford B thoracic aortic dissection. *Intern Med Chin*. 2008,3(5):704-705.

69. Wang S, Ye J, Chang G, Chen X, Li J, et al. Endovascular graft exclusion for the treatment of Stanford B aortic aneurysm. *Chin Arch Gen Surg*. 2008,2(5):394-395.

70. Sui X. Endovascular graft exclusion of Stanford B aortic dissection aneurysm in 20 patients. *Clin Focus*. 2008,23(17):1254-1255.

71. Zhang W, Sun J, Zhang H, Li S, Zhu J, et al. Clinical observation of Chinese endovascular stent implantation of Stanford B aortic dissection in elderly patients. *Chin J Gerontol*. 2008,28(11):1103-1104.

72. Zhang X, Xie X, Liu H, Li J, Xiao B. Nursing care of endovascular stent graft exclusion for the treatment of Debakey III aortic dissection. *J Qiqihar Univ Med*. 2011,32(7):1156.

73. Zhang C, Li Z, Liu R, Zhang D, Li L. Comparison of Anesthetic Effectiveness between Three Anesthesia Methods for DeBakey III Aortic Dissecting Patients during Covered Stented Implantation. *J Med Forum*. 2008,29(24):43-46.

74. Che Z, Wu J, Yang S, Gao P, Wu X, et al. Endovascular Repair of Descending Aorta Dilatation. *Chin J Min Inv Surg*. 2012,12(5):393-395.

75. Zhang J, Li Q, Yuan Y, Luo W, Cheng D, et al. Diagnosis and treatment cases of aortic vascular disease in 14 cases. *PJCCPVD*. 2010,18(12):1864-1865.

76. Yang C, Li J, Wu W, Xu Z, Wang Q, et al. The value of endovascular stent graft repair in the treatment of aortic diseases. *Chin Imaging J Integr Tradit West Med*.2010,8(3):231-234.

77. Hao L. Report of 11 cases of endovascular graft exclusion of Stanford type B aortic dissection aneurysm by stent graft implantation. *Inner Mongolia Med J*. 2008,40(10):1241-1242.

78. Zhou Z, Wang Z, Li G, Zhang S, Yu Z. Evaluation of clinic for endovascular stent grafting treatment the thoracic aortic dissection and abdominal aortic aneurysm. *Chin Imaging J Integr Tradit West Med*. 2008,6(4):273-275.

79. He P, Luo J, Luo S, Huang W, Liu Y, et al. Prevalence and impact of concomitant coronary artery disease in aged patients with Stanford type B aortic dissection. *Chin J Thorac Cardiovasc Surg*. 2014,30(9):535-538.

80. Xing X, Chen J, Gu X, Chen C. Endovascular aortic repair combined with fenestrated stent-grafts techniques for the treatment of Stanford type B aortic dissection involving aortic arch. *J Clin Surg*. 2014,22(7):492-494.

81. Li J, Quan H, Xue K, Yang J. Experience of Endovascular graft exclusion for acute Stanford B aortic dissection by local anesthesia in 40 cases. *Jilin Med J*. 2014,35(19):4167-4170.

82. Wei J, Su X, Song D, Peng J, Yan H, et al. Effects of endovascular placement of stent-grafts in the prognosis of Stanford type B aortic dissection. *Chin J Cardiovasc Res*. 2014,12(7):608-671.

83. Ma Y, Qi Y, Yuan Y, Li Q, Zhou W. Experience in the treatment of 22 cases of Stanford B aortic dissection aneurysm. *Gansu Med J*.2014,33(6):427-430.

84. Yang J, Xiang D, Xiao H, Peng D, Zhang J. Investigate on the correlations between the time of thoracic endovascular aortic repair and prognosis in patients with acute Stanford type B aortic dissection. *Chin J Interventional Cardiol*. 2014,22(5):300-303.

85. Liu R, Yang J, Chen J, Nie R. One-stage Hybrid Surgery for Complex Stanford Type B Aortic Dissection. *Chin J Clin Thorac Cardiovasc Surg*. 2014,21(3):352-355.

86. Wang X, Li G, Yang J. Outcomes and experience of thoracic endovascular aortic repair for Stanford type B aortic dissection. *South China J Cardiovasc Dis*. 2014,20(4):480-482.

87. Yin L, Song B, Guan Y, Li Y, Chen G, et al. Clinical value of dual -source CT angiography in diagnosis and following-up observation after endovascular exclusion of Stanford type B aortic dissection. *J Pract Radiol*. 2014,30(7):1125-1128.

88. Hu Y, Wang Z, Hu X. Therapy choice for Stanford type B aortic dissection. *Chin J Gen Surg*. 2014,29(7):538-541.

89. Gou X, Li Q. Efficiency of Endovascular Graft Exclusion for the Treatment of Stanford Type B Aortic Dissection. *Chin Med Innovat*. 2014,11(11):136-138.

90. Jin W, Lu S, Yang M, Wang W, Fan J. Clinical analysis of endovascular grant exclusion for 46 patients with Stanford type B aortic dissection. *Chin J Postgrad Med*. 2014,37(2):29-31.

91. Zhang Y, Liang J, Zheng X, Zhang B. Experience of endovascular exclusion for type B aortic dissection in 36 cases. *Chin J Postgrad Med*. 2012,35(11):65-67.

92. Pan L, Huang J, Ni H, Zheng X, Su X, et al. Treatment of Stanford B aortic dissection with aberrant right clavicle artery. *Zhejiang Clin Med*. 2013,15(12):1826-1827.

93. Yang Y, Gu W, Li W, Zhang X, Shen C, et al. Thoracic endovascular aortic repair for chronic type B aortic dissection in 30 patients. *Chin J Gen Surg*. 2013,28(12):918-921.

94. Zhou W, Zhou W, Qiu J, Yuan Q, Chen F, et al. Endovascular treatment of malperfusion in acute type B aortic dissections. *Chin J Gen Surg*. 2013,28(8):572-575.

95. Tang W, Liu Y, Zhang J, Feng W. Endovascular stent graft exclusion for the treatment of type B aortic dissection in 24 cases. Chin J Pract Med. 2013,40(14):102-103.

96. Qiu F, Li L. Clinical observation fo endovascular stent graft exclusion for the treatment of Stanford B aortic dissection in 33 patients. *China Pract Med*. 2013,8(23):97-98.

97. Yu G, Wang Z, Huang B, Chen G, Wu S, et al. Application of covered stent in Stanford B thoracic aortic dissection. *Jiangsu Med J.* 2012,38(12):1471-1472.

98. Jiang Z, Mei J, Ding F, Bao C, Tang M, et al. Clinical experience of early anticoagulant therapy after endovascular stent-graft exclusion for Stanford B type aortic dissection. *Chin J Thorac Cardiovasc Surg*. 2012,28(6):324-327.

99. Tang J, Huang J, Zuo K, Qian Z, Hang W, et al. Endovascular stent-grafts for acute Stanford type B aortic dissection. *Chin J Gen Surg*. 2010,25(3):221-223.

100. Xiao Y, Mao J, Shi S, Xiao M. Endovascular Stenting for the Treatment of Thoracic Aortic Aneurysm. *Chin J Min Inv Surg*. 2009,9(5):388-390.

101. Mo W, Wang W, Zhou X, Shen J, Wang K. Clinical observation of endovascular exclusion in treatment of elderly patients with acute stanford type B aortic dissection. *J Crit Care Intern Med*. 2014,20(4):251-252.

102. Cheng S, Xue S, Tang M, Zhai X, Hu Z, et al. Endovascular exclusion of Stanford B dissecting aneurysm. *J Shanghai Jiaotong Univ*. 2009,29(6):758-759.

103. Zhao Q, Chen K, Jiang Z, Sang H. Analysis of the effect of endovascular graft exclusion for the treatment of De Bakey III aortic dissection. *Henan Med Res*. 2014,23(4):61-63.

104. Bi P. Comfortable nursing application in patients with DeBakey Ⅲ aortic dissection after carotid stenting. *Chin Med Herald*. 2014,11(23):116-119.

105. Luo Y, Liu Y, Sheng G. Clinical analysis of DeBakey type III aortic dissection in 21 cases. *South China J Cardiovasc Dis*.2014,20(2):213-214, 222 .

106. Tang Y, Wang H, Yue L, Wen S, Li H. Endovascular stent graft exclusion for the treatment of aortic dissection in 10 cases. *Heilongjiang Med J*. 2013,26(6):1079-1081.

107. Xi E, Zhu J, Zhu S, Zhang Y, Xu G, et al. The Clinical Studying of Endovascular Aortic Ｒepair for Acute and Chronic Aortic Dissection. *Chin J Gen Prac*. 2013,11(5):686-688.

108. Jin Z, Li J, Du D. Clinical research on endovascular graft exclusion in treatment of DeBakey type III dissecting aneurysm. *Mod Chin Doc.* 2013,51(25):119-120.

109. Zhou T, Xu B, Shen T. Aortic remodeling after endovascular stent-grafts for DeBakey Ⅲ type aortic dissection. *J Tongji Univ*. 2013,34(3):81-85.

110. Cao H, Jiang D, Chen L. Endovascular stent graft exclusion for the treatment experience of Debakey III aortic dissection in 60 cases. *Jilin Med J*. 2012,33(8):1627-1629.

111. Li X, Zuo W, Fu Y, Zhu F, Dong X, et al. Application of endovascular stent-graft implantation for the treatment of DeBakey type III aortic dissection. *Chin J Interv Imaging Ther.* 2012,9(7):564-565.

112. Yao Y. Perioperative nursing care of endovascular graft exclusion for Debakey III aortic dissection in 6 cases. *J Bengbu Med Coll*. 2012,37(12):1542-1543.

113. Lin J, Zhu L, Yin Y, Ruan Z. Endovascular graft exclusion in the treatment of Debakey III aortic dissection with domestic-made stent-graft in 20 cases. *J Clin Med Prac*. 2012,16(22):33-35.

114. Liu Y, Yue X. Perioperative health education in 15 patients with type III Aortic Dissection Stent Intervention Surgery. *Chin Med Eng*. 2012,20(11):117.

115. Li C, Han T, Ding H, Yao Z, Chen L, et al. A follow-up study of endovascular exclusion for Debakey III aortic dissection in 55 cases. *J Fujian Med Univ*. 2012,46(6):433-435.

116. Zhu J, Yao Z, Guo Y, Ma L. Experience on endovascular repair of DeBakey III aortic dissection: a report of 15 cases. *Hainan Med J*. 2012,23(7):34-35.

117. Liu K, Dong G, Xu B, Jing H, You Q, et al. Comparison of curative effects of endovascular graft exclusion for acute and chronic type DeBakey III aortic dissection. *Chin J Crit Care Med*. 2009,29(8):688-691.

118. Li P, Liu H, Yang H. Operative coordination of stent implantation surgery of 9 cases under local anesthesia for DeBaKey III aortic dissection. *Med Inf*. 2011,24(4):49.

119. Xu K, Wu X, Chen R, Xiao L, Zhang X, et al. Evaluation of the effect on endovascular repair of DeBakey m aortic dissection: Mid-term follow-up result. *J Intervent Radiol*. 2008,17(8):567-569.

120. Han B, Zhang H, Zhou C, Ji G, Yang Z, et al. Clinical analysis of 56 cases in DeBakey type III aortic dissection aneurysm with endovascular graft exclusion. *J Hebei Med Coll Contin Edu*. 2001,25(5):7-9.

121. Zhang Z, Yang B. Endovascular Stent-Graft Placement for the Treatment of Debakey III Type Aortic Dissections. *Chin J Min Inv Surg*. 2008,8(1):70-71.

122. Yang C, Su Y, Zhang X, Zhang X, Lu H. A study on peri—operative nursing care of patients with dissection of aorta of Debakey type III treated with covered stent. *Chin Nurs Res*. 2008,22(1):238-239.

123. Lu Z, Zhang J, Zhou Y, Meng J. Percutaneous endovascular stent graft exclusion for the treatment of DeBakey III aortic dissection. *PJCCPVD*. 2011,19(6):993.

124. Luo H. The techniques and effects of percutaneous endovascular stent graft exclusion for treatment of aortic dissection aneurysm (Debakey III). *J Chin Tradit Chin Med Inf*. 2011,3(19):99-100.

125. Huai Z, Li X, Zhang J. Nursing experience of endovascular stent implantation for type III aortic dissection aneurysm in 25 cases. *Seek Med Ask Med*. 2011,9(9):153.

126. Sun Y, Huang B, Yu J, Zhuang M, Li B. Treatment of internal leakage after endovascular exclusion for Debakey type III aortic dissection. *PJCCPVD*. 2011,19(3):414-415.

127. Yang B. Clinical analysis of transluminal stent-graft placement for thoracic aortic dissections. *Anhui Med J.* 2011,32(4):468-470.

128. Guo Q. Nursing experience of endovascular stent graft exclusion for aortic dissection in 9 cases. *J Baotou Med Coll*. 2008,24(4):408-409.

129. Wang C, Xu Y, Cheng Z, Guo J, Wu M. Clinical observation of endovascular graft exclusion in the treatment of De Bakey III aortic dissection aneurysm. *Acta Univ Med Anhui*. 2011,46(1):97-98.

130. Wei W, Wu J, Ji W, Han X, Yang D. Endovascular interventional treatment of Debakey type III aortic dissection aneurysm in 11 patients. *Jilin Med J*. 2010,31(28):4853-4854.

131. Wang J, Li C. Treatment of Debakey III aortic dissection with domestic branched endovascular stent-graft. *J Clin Cardiol*. 2010,26(11):824-826.

132. Huang X, Zeng Z. Nursing care of endovascular stent graft exclusion for the treatment of Debakey III aortic dissection aneurysm. *Chin J Pract Nurs*. 2010,26(1):27-29.

133. Liao W, Cai J, Zhou A. Improved endovascular stent-graft exclusion for Debakey III type aortic dissections. *J Clin Rehabilit Tiss Eng Res*. 2009,13(48):9483-9486.

134. Wu Q, Huang J, Huang J, Xu G. Effect of Transluminal Stent-Graft Placement For Debakey III Type Aortic Dissection Aneurysm. *Chin J Gen Prac*. 2009,7(8):834-835.

135. Hu G, Jin B, Zheng H, Lai C, Ouyang C, et al. Analysis of 287 Patients with Aortic Dissection: General Characteristics, Outcomes and Risk Factors in a Single Center. *J Huazhong Univ Sci Technol*. 2011,31(1):107-113.

136. Liu J, Jiang W, Lu H, Li Y, Zhang T, et al. Application of Protective Stents in Endovascular Repair of Acute Complicated Stanford Type B Aortic Dissections. *J Endovasc Ther*. 2013,20:210–218.

137. Xu S, Huang F, Yang J, Li Z, Yang S, et al. Early and midterm results of thoracic endovascular aortic repair of chronic type B aortic dissection. *J Thorac Cardiovasc surg*. 2010,139(6):1548-1553.

138. Chang G, Li X, Chen W, Li J, Hu Z, et al. Early to midtem results of endovascular repair of aortic dissection：report of 165 cases. *Chin J Surg*. 2008,46(10):752-755.

139. Li Q, Huang L, Xu S, Zhu J, Liu Y, et al. Effects of thoracic endovascular repair in patients of type B aortic dissection. *Natl Med J China*. 2013,93(19):1469-1471.

140. Yu D, Yan J, Zeng H. Fever after Percutaneous Endovascular Stent-graft in Type B Acute Aortic Dissection. *Intern Med*. 2011,50:2095-2098.

141. Feng J, Lu Q, Zhao Z, Bao J, Feng X, et al. Restrictive bare stent for prevention of stent graft-induced distal redissection after thoracic endovascular aortic repair for type B aortic dissection. *J Vascular Surg*. 2013,57(2):44-52.

142. Qin Y, Deng G, Li T, Wang W, Teng G. Treatment of Acute Type-B Aortic Dissection. *Cardiovasc Intervent*. 2013,6(2):185-191.

143. Mao X. Observation and nursing care of Endovascular graft exclusion of elderly patients with type B aortic dissection. *J North pharm.* 2014,11(2):144-145.

144. Jin T, Jin L, Zhang H, Huang L. Nursing care of elderly patients with Stanford B aortic dissection undergoing stent implantation. *Chin J Tiss Eng Res*. 2014,(zl):117.

145. Chen L. Perioperative nursing care of acute B type Stanford aortic dissection treated with thoracic aortic endovascular repair in 36 patients. *J Qilu Nurs.* 2013,19(24):115-116.

146. Jiang R, Ji S, Wang J, Ding H, Zheng Y. Perioperative nursing care of Standford B aortic dissection treated with endovascular stent graft in 61 cases. *J Qilu Nurs*. 2013,19(20):105-107.

147. Wang Y. Perioperative nursing care of patients with Stanford B aortic dissection. *Med Inf*. 2011,24(9):4856-4857.

148. Wang J. Clinic Study of Endovascular Repair on Stanford Type B Aortic Dissection. Ph.D.Thesis, *Southern Medical University*, 2012.

149. Li J, Dou N, Zhang Y, Fang G, Ma Y, et al. Comparison of endovascular graft exclusion of subacute and chronic Stanford B aortic dissection. *Ningxia Med J*. 2012,34(9):869-871.

150. Zhang Y, Huo X. Endovascular repair for thoracic dissecting aneurysm. *Chin J Gen Surg*. 2009,18(12):1238-1240.

151. Dong Y, Cao W, Lu A, Dong S, Yin Q, et al. Short-term curative effect of endovascular repair in treatment of type B aortic dissection. *J Cardiovascular Pulmonary Dis*. 2011,30(6):524-527.

152. Fan H. Perioperative nursing care of DeBakey III aortic dissection treated with covered stent implantation. *Exam Week.* 2014,(8):193-194.

153. Nong J. Follow-up and clinical study of aortic remodeling in stanford type B aortic dissection with endovascular graft exclusion. *Master Thesis, Guangxi Medical University*, 2012.

154. Zhang M. Analysis of Stanford B aortic dissection in 117 cases. *Master Thesis,Central South University*, 2011.

155. Gong K, Xiao L, Wang K, Ouyang Y, Ling P, et al. Endovascular revascularization for visceral ischemia in patients of type B dissection. J Cardiovascular Pulmonary Dis. 2010,29(6):465-466.

156. Jia Y, Li J, Su Y, Ma J, Guan X, et al. Midterm results of thoracic aortic dessection endovascular repair in conjunctions with the location of Adamkiewicz artery. *Natl Med J China*. 2012,92(39):2752-2755.

157. Shu C, Fang K, Luo M, Li Q, Wang Z. Emergency endovascular stent-grafting for acute type B aortic dissection with symptomatic malperfusion. *Int Angiol*. 2013,32(5):483-491.

158. Dong Z, Fu W, Wang Y, Guo D, Xu X, et al. Retrograde Type A Aortic Dissection After Endovascular Stent Graft Placement for Treatment of Type B Dissection. *Circulation*. 2009,119:735-741.

159. Bing X. Endovascular stent graft repair for the treatment of Stanford B aortic dissection aneurysm. *Shandong Med J*. 2013,53(12):39-40.

160. Ge Y. Correlation between preoperative thoracic side branches arising from False Lumen(tSAFL) and aortic remodeling after TEVAR in Debakey IIIb aortic dissection. *Master Thesis,Medical School of Chinese PLA*. 2015.

161. Yang F, Wang J, Long C, Tong Y, Sun H, et al. Evaluation of endovascular covered- stent implantation in treating Stanford type B aortic dissection. *J Interventional Radiol.* 2015,24(3):197-199.

162. Guo B. Clinical analysis of endovascular repair for 32 patienfs with Stanford type B aortic dissection. *Chin J Clin Rational Drug Use*. 2015,8(2):104-105.

163. Jiang L, Wang X, Bai D, Wu S, Zhang X, et al. On Establishing Track Delivery in Endovascular Treatment of Thoracic Aortic Dissection. *Chin J Min Inv Surg*. 2015,15(2):101-103.

164. Wang Q, Xiang H, Yan P, Zhang Z, Liu J, et al. TEVAR Therapy for Stanford Type B Aortic Dissection. *J Hunan Normal Univ.* 2014,11(4):43-46.

165. Feng J, Ge S, Zhou R, Li F, Zhang S, et al. "One-step" hybrid operation and simple endovascular graft exclusion for the treatment of Stanford B aortic dissection. *Anhui Med J*. 2015,36(4):476-478.

166. Bo Q, Lv M. Nursing cooperation of 122 cases of B Stanford aortic dissection repair in hybrid operation room. *J Qilu Nurs*. 2014,20(24):69-70.

167. Chen C, Tu H, Yan C. Short-term clinical outcome of endovascular covered stent grafting in early stage in patients with type III aortic dissection. *South China J Cardiovasc Dis.* 2014,20(6):722-723.

168. Xiong J. A single-center’s experience on clinical applyment of tapered design stentgraft in the treatment of aortic dissection. *Chin Foreign Med Treat.* 2015,(5):104-106.

169. Zhou M, Zhu Y, Zhu Z, Li Z, Zhang S, et al. Factors of proximal type I endoleak after thoracic endovascular aortic repair in patients with DeBakey type III aortic dissection. *South China J Cardiovasc Dis*. 2014,20(6):729-733.

170. Zhao L, Zhang J, Zhang L, Liu P. Early efficacy analysis and perioperative management of endovascular repair of Stanford B aortic dissection. *J China-Japan Friendship Hosp*. 2014,28(6):345-346 349.

171. Fan B, Chen C. Endovascular stent graft exclusion for the treatment of aortic dissection in 87 cases. *Chin J Surg Integr Tradit West Med*. 2014,20(5):522-524.

172. Cai L, Cai M, Chen Q, Lin X, Xu L, et al. The Treatment of Type B Aortic Dissection:a Report of 52 Cases. *Chin J Min Inv Surg*. 2013,13(10):899-902.

173. Li D. Analysis of mid term outcome of endovascular repair for Stanford B aortic dissection and aortic morphological remodeling. *Ph.D.Thesis, Zhejiang University*, 2014.

174. Tan G. Endovascular treatment of Stanford B aortic dissection and mid term follow up of postoperative aortic remodeling. *Master Thesis, Dalian Medical University*, 2014.

175. Qiao Q. Comparison of mid term efficacy of endovascular aortic repair and drug treatment for Stanford type B dissection. *Master Thesis, Hebei Medical University,* 2014.
